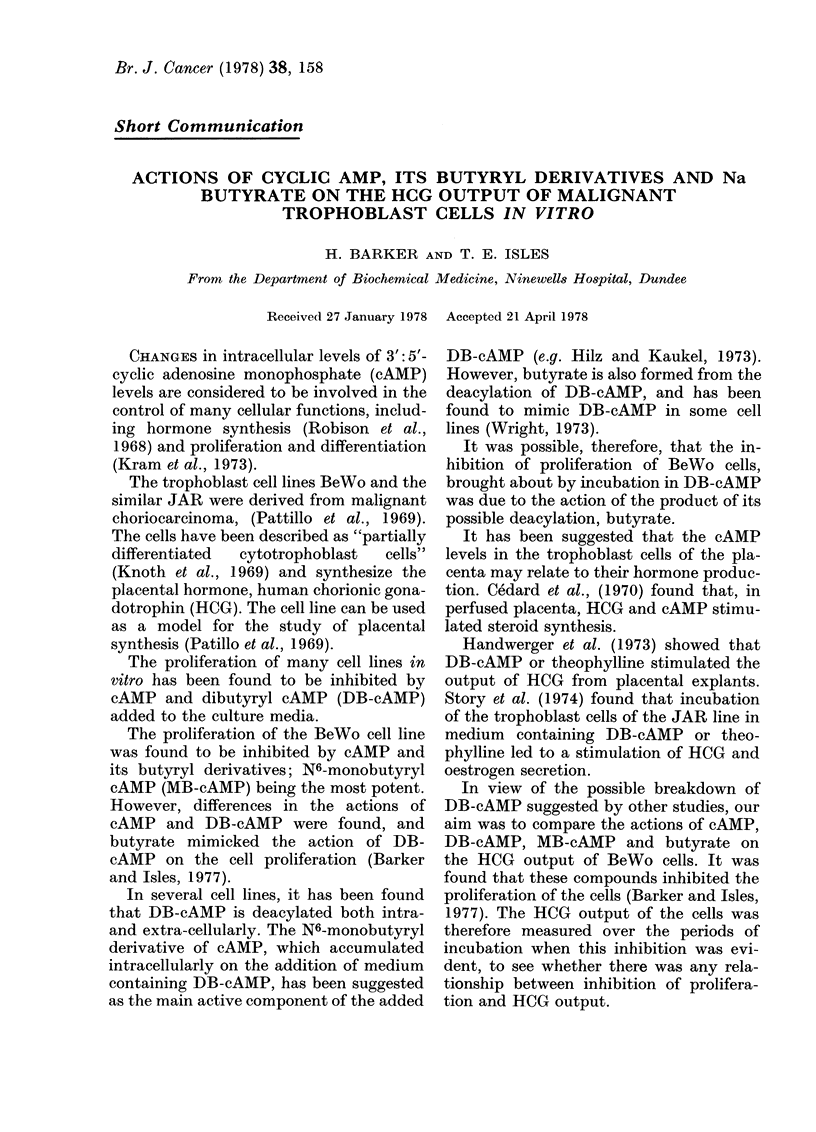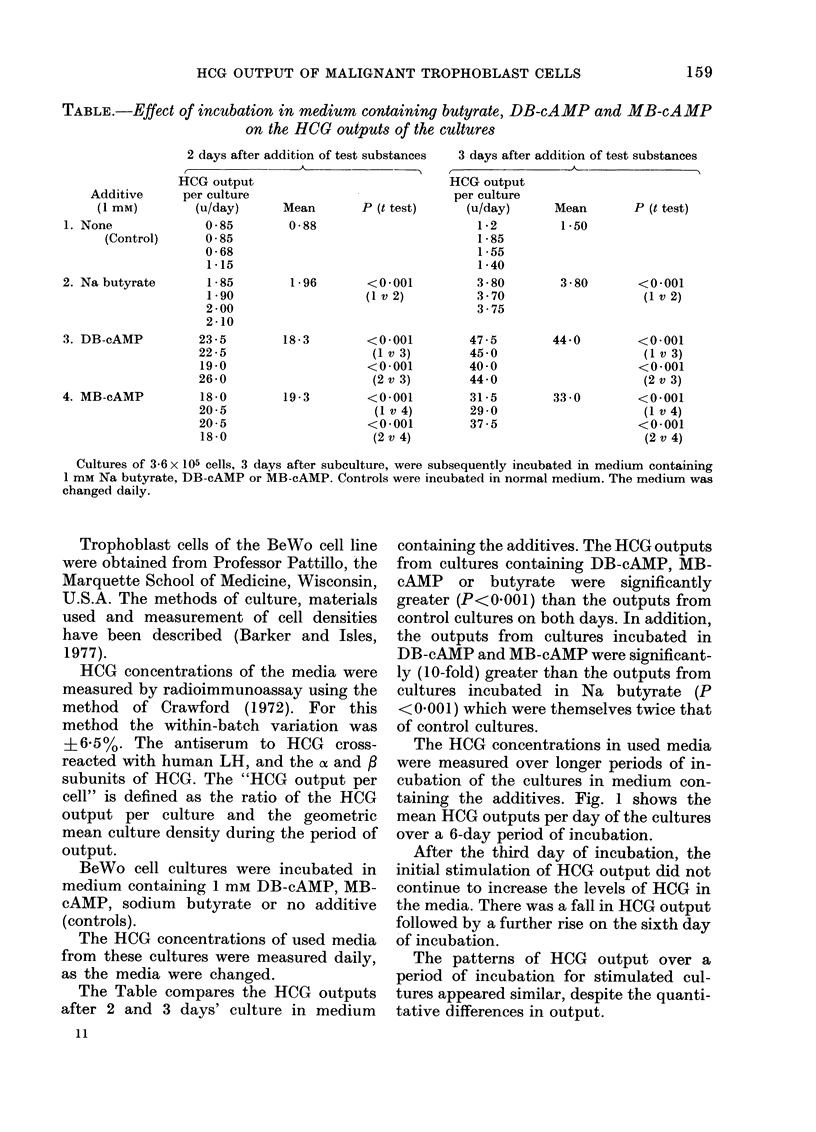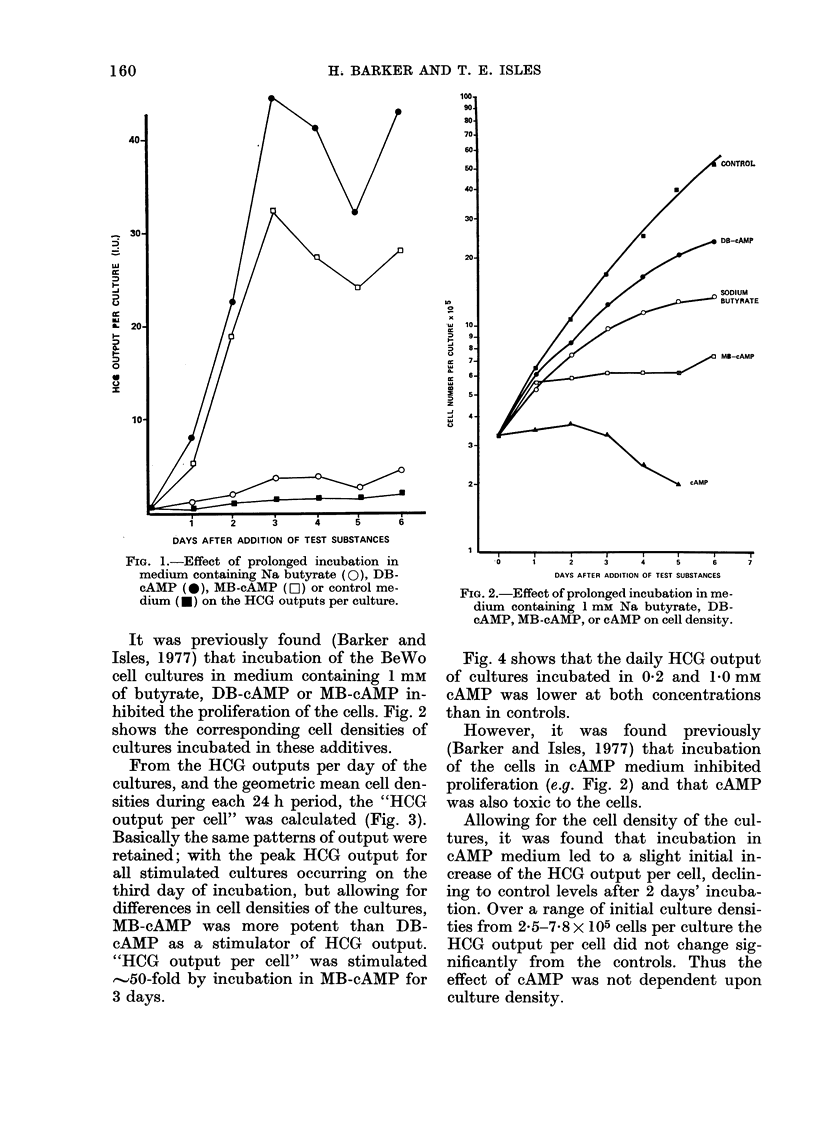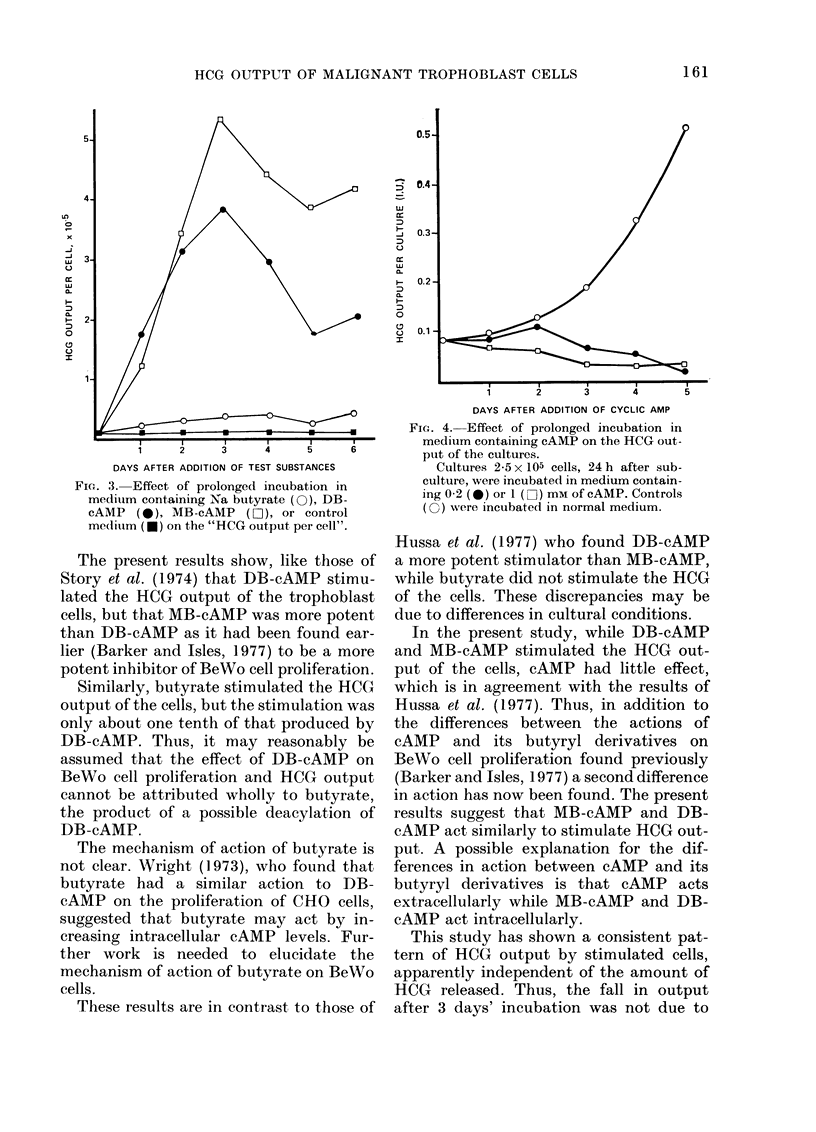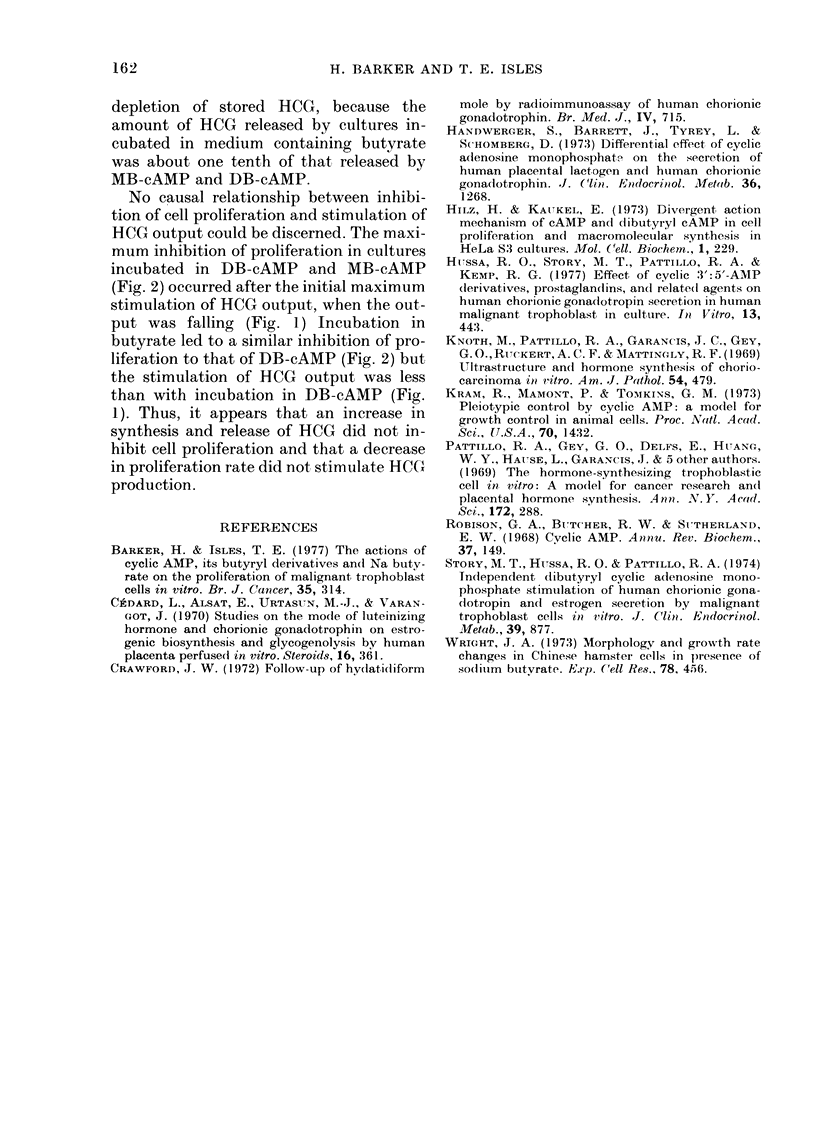# Actions of cyclic AMP, its butyryl derivatives and Na butyrate on the HCG output of malignant trophoblast cells in vitro.

**DOI:** 10.1038/bjc.1978.178

**Published:** 1978-07

**Authors:** H. Barker, T. E. Isles


*Br. J. Cancer* (1978) **38**, 158

***Short Communication***

### ACTIONS OF CYCLIC AMP, ITS BUTYRYL DERIVATIVES AND Na BUTYRATE ON THE HCG OUTPUT OF MALIGNANT TROPHOBLAST CELLS *IN VITRO*

H. BARKER AND T. E. ISLES

*From the Department of Biochemical Medicine, Ninewells Hospital, Dundee*

Received 27 January 1978 Accepted 21 April 1978

CHANGES in intracellular levels of 3′:5′-cyclic adenosine monophosphate (cAMP) levels are considered to be involved in the control of many cellular functions, including hormone synthesis (Robison *et al.*, 1968) and proliferation and differentiation (Kram *et al.*, 1973).

The trophoblast cell lines BeWo and the similar JAR were derived from malignant choriocarcinoma, (Pattillo *et al.*, 1969). The cells have been described as "partially differentiated cytotrophoblast cells" (Knoth *et al.*, 1969) and synthesize the placental hormone, human chorionic gonadotrophin (HCG). The cell line can be used as a model for the study of placental synthesis (Patillo *et al.*, 1969).

The proliferation of many cell lines *in vitro* has been found to be inhibited by cAMP and dibutyryl cAMP (DB-cAMP) added to the culture media.

The proliferation of the BeWo cell line was found to be inhibited by cAMP and its butyryl derivatives; $N^6$-monobutyryl cAMP (MB-cAMP) being the most potent. However, differences in the actions of cAMP and DB-cAMP were found, and butyrate mimicked the action of DB-cAMP on the cell proliferation (Barker and Isles, 1977).

In several cell lines, it has been found that DB-cAMP is deacylated both intra- and extra-cellularly. The $N^6$-monobutyryl derivative of cAMP, which accumulated intracellularly on the addition of medium containing DB-cAMP, has been suggested as the main active component of the added DB-cAMP (*e.g.* Hilz and Kaukel, 1973). However, butyrate is also formed from the deacylation of DB-cAMP, and has been found to mimic DB-cAMP in some cell lines (Wright, 1973).

It was possible, therefore, that the inhibition of proliferation of BeWo cells, brought about by incubation in DB-cAMP was due to the action of the product of its possible deacylation, butyrate.

It has been suggested that the cAMP levels in the trophoblast cells of the placenta may relate to their hormone production. Cédard *et al.*, (1970) found that, in perfused placenta, HCG and cAMP stimulated steroid synthesis.

Handwerger *et al.* (1973) showed that DB-cAMP or theophylline stimulated the output of HCG from placental explants. Story *et al.* (1974) found that incubation of the trophoblast cells of the JAR line in medium containing DB-cAMP or theophylline led to a stimulation of HCG and oestrogen secretion.

In view of the possible breakdown of DB-cAMP suggested by other studies, our aim was to compare the actions of cAMP, DB-cAMP, MB-cAMP and butyrate on the HCG output of BeWo cells. It was found that these compounds inhibited the proliferation of the cells (Barker and Isles, 1977). The HCG output of the cells was therefore measured over the periods of incubation when this inhibition was evident, to see whether there was any relationship between inhibition of proliferation and HCG output.

TABLE.—*Effect of incubation in medium containing butyrate, DB-cAMP and MB-cAMP on the HCG outputs of the cultures*

| Additive (1 mM) | 2 days after addition of test substances: HCG output per culture (u/day) | Mean | P (t test) | 3 days after addition of test substances: HCG output per culture (u/day) | Mean | P (t test) |
|---|---|---|---|---|---|---|
| 1. None (Control) | 0·85 | 0·88 | | 1·2 | 1·50 | |
| | 0·85 | | | 1·85 | | |
| | 0·68 | | | 1·55 | | |
| | 1·15 | | | 1·40 | | |
| 2. Na butyrate | 1·85 | 1·96 | <0·001 | 3·80 | 3·80 | <0·001 |
| | 1·90 | | (1 v 2) | 3·70 | | (1 v 2) |
| | 2·00 | | | 3·75 | | |
| | 2·10 | | | | | |
| 3. DB-cAMP | 23·5 | 18·3 | <0·001 | 47·5 | 44·0 | <0·001 |
| | 22·5 | | (1 v 3) | 45·0 | | (1 v 3) |
| | 19·0 | | <0·001 | 40·0 | | <0·001 |
| | 26·0 | | (2 v 3) | 44·0 | | (2 v 3) |
| 4. MB-cAMP | 18·0 | 19·3 | <0·001 | 31·5 | 33·0 | <0·001 |
| | 20·5 | | (1 v 4) | 29·0 | | (1 v 4) |
| | 20·5 | | <0·001 | 37·5 | | <0·001 |
| | 18·0 | | (2 v 4) | | | (2 v 4) |

Cultures of $3 \cdot 6 \times 10^5$ cells, 3 days after subculture, were subsequently incubated in medium containing 1 mM Na butyrate, DB-cAMP or MB-cAMP. Controls were incubated in normal medium. The medium was changed daily.

Trophoblast cells of the BeWo cell line were obtained from Professor Pattillo, the Marquette School of Medicine, Wisconsin, U.S.A. The methods of culture, materials used and measurement of cell densities have been described (Barker and Isles, 1977).

HCG concentrations of the media were measured by radioimmunoassay using the method of Crawford (1972). For this method the within-batch variation was $\pm 6 \cdot 5\%$. The antiserum to HCG cross-reacted with human LH, and the $\alpha$ and $\beta$ subunits of HCG. The "HCG output per cell" is defined as the ratio of the HCG output per culture and the geometric mean culture density during the period of output.

BeWo cell cultures were incubated in medium containing 1 mM DB-cAMP, MB-cAMP, sodium butyrate or no additive (controls).

The HCG concentrations of used media from these cultures were measured daily, as the media were changed.

The Table compares the HCG outputs after 2 and 3 days' culture in medium containing the additives. The HCG outputs from cultures containing DB-cAMP, MB-cAMP or butyrate were significantly greater ($P < 0 \cdot 001$) than the outputs from control cultures on both days. In addition, the outputs from cultures incubated in DB-cAMP and MB-cAMP were significantly (10-fold) greater than the outputs from cultures incubated in Na butyrate ($P < 0 \cdot 001$) which were themselves twice that of control cultures.

The HCG concentrations in used media were measured over longer periods of incubation of the cultures in medium containing the additives. Fig. 1 shows the mean HCG outputs per day of the cultures over a 6-day period of incubation.

After the third day of incubation, the initial stimulation of HCG output did not continue to increase the levels of HCG in the media. There was a fall in HCG output followed by a further rise on the sixth day of incubation.

The patterns of HCG output over a period of incubation for stimulated cultures appeared similar, despite the quantitative differences in output.

FIG. 1.—Effect of prolonged incubation in medium containing Na butyrate (○), DB-cAMP (●), MB-cAMP (□) or control medium (■) on the HCG outputs per culture.

FIG. 2.—Effect of prolonged incubation in medium containing 1 mM Na butyrate, DB-cAMP, MB-cAMP, or cAMP on cell density.

It was previously found (Barker and Isles, 1977) that incubation of the BeWo cell cultures in medium containing 1 mM of butyrate, DB-cAMP or MB-cAMP inhibited the proliferation of the cells. Fig. 2 shows the corresponding cell densities of cultures incubated in these additives.

From the HCG outputs per day of the cultures, and the geometric mean cell densities during each 24 h period, the "HCG output per cell" was calculated (Fig. 3). Basically the same patterns of output were retained; with the peak HCG output for all stimulated cultures occurring on the third day of incubation, but allowing for differences in cell densities of the cultures, MB-cAMP was more potent than DB-cAMP as a stimulator of HCG output. "HCG output per cell" was stimulated ~50-fold by incubation in MB-cAMP for 3 days.

Fig. 4 shows that the daily HCG output of cultures incubated in 0·2 and 1·0 mM cAMP was lower at both concentrations than in controls.

However, it was found previously (Barker and Isles, 1977) that incubation of the cells in cAMP medium inhibited proliferation (*e.g.* Fig. 2) and that cAMP was also toxic to the cells.

Allowing for the cell density of the cultures, it was found that incubation in cAMP medium led to a slight initial increase of the HCG output per cell, declining to control levels after 2 days' incubation. Over a range of initial culture densities from 2·5–7·8 $\times 10^5$ cells per culture the HCG output per cell did not change significantly from the controls. Thus the effect of cAMP was not dependent upon culture density.

FIG. 3.—Effect of prolonged incubation in medium containing Na butyrate (○), DB-cAMP (●), MB-cAMP (□), or control medium (■) on the "HCG output per cell".

FIG. 4.—Effect of prolonged incubation in medium containing cAMP on the HCG output of the cultures.

Cultures $2{\cdot}5 \times 10^5$ cells, 24 h after subculture, were incubated in medium containing 0·2 (●) or 1 (□) mM of cAMP. Controls (○) were incubated in normal medium.

The present results show, like those of Story *et al.* (1974) that DB-cAMP stimulated the HCG output of the trophoblast cells, but that MB-cAMP was more potent than DB-cAMP as it had been found earlier (Barker and Isles, 1977) to be a more potent inhibitor of BeWo cell proliferation.

Similarly, butyrate stimulated the HCG output of the cells, but the stimulation was only about one tenth of that produced by DB-cAMP. Thus, it may reasonably be assumed that the effect of DB-cAMP on BeWo cell proliferation and HCG output cannot be attributed wholly to butyrate, the product of a possible deacylation of DB-cAMP.

The mechanism of action of butyrate is not clear. Wright (1973), who found that butyrate had a similar action to DB-cAMP on the proliferation of CHO cells, suggested that butyrate may act by increasing intracellular cAMP levels. Further work is needed to elucidate the mechanism of action of butyrate on BeWo cells.

These results are in contrast to those of Hussa *et al.* (1977) who found DB-cAMP a more potent stimulator than MB-cAMP, while butyrate did not stimulate the HCG of the cells. These discrepancies may be due to differences in cultural conditions.

In the present study, while DB-cAMP and MB-cAMP stimulated the HCG output of the cells, cAMP had little effect, which is in agreement with the results of Hussa *et al.* (1977). Thus, in addition to the differences between the actions of cAMP and its butyryl derivatives on BeWo cell proliferation found previously (Barker and Isles, 1977) a second difference in action has now been found. The present results suggest that MB-cAMP and DB-cAMP act similarly to stimulate HCG output. A possible explanation for the differences in action between cAMP and its butyryl derivatives is that cAMP acts extracellularly while MB-cAMP and DB-cAMP act intracellularly.

This study has shown a consistent pattern of HCG output by stimulated cells, apparently independent of the amount of HCG released. Thus, the fall in output after 3 days' incubation was not due to depletion of stored HCG, because the amount of HCG released by cultures incubated in medium containing butyrate was about one tenth of that released by MB-cAMP and DB-cAMP.

No causal relationship between inhibition of cell proliferation and stimulation of HCG output could be discerned. The maximum inhibition of proliferation in cultures incubated in DB-cAMP and MB-cAMP (Fig. 2) occurred after the initial maximum stimulation of HCG output, when the output was falling (Fig. 1) Incubation in butyrate led to a similar inhibition of proliferation to that of DB-cAMP (Fig. 2) but the stimulation of HCG output was less than with incubation in DB-cAMP (Fig. 1). Thus, it appears that an increase in synthesis and release of HCG did not inhibit cell proliferation and that a decrease in proliferation rate did not stimulate HCG production.

### REFERENCES

BARKER, H. & ISLES, T. E. (1977) The actions of cyclic AMP, its butyryl derivatives and Na butyrate on the proliferation of malignant trophoblast cells *in vitro*. *Br. J. Cancer*, **35**, 314.

CÉDARD, L., ALSAT, E., URTASUN, M.-J., & VARANGOT, J. (1970) Studies on the mode of luteinizing hormone and chorionic gonadotrophin on estrogenic biosynthesis and glycogenolysis by human placenta perfused *in vitro*. *Steroids*, **16**, 361.

CRAWFORD, J. W. (1972) Follow-up of hydatidiform mole by radioimmunoassay of human chorionic gonadotrophin. *Br. Med. J.*, **IV**, 715.

HANDWERGER, S., BARRETT, J., TYREY, L. & SCHOMBERG, D. (1973) Differential effect of cyclic adenosine monophosphate on the secretion of human placental lactogen and human chorionic gonadotrophin. *J. Clin. Endocrinol. Metab.* **36**, 1268.

HILZ, H. & KAUKEL, E. (1973) Divergent action mechanism of cAMP and dibutyryl cAMP in cell proliferation and macromolecular synthesis in HeLa S3 cultures. *Mol. Cell. Biochem.*, **1**, 229.

HUSSA, R. O., STORY, M. T., PATTILLO, R. A. & KEMP, R. G. (1977) Effect of cyclic 3′:5′-AMP derivatives, prostaglandins, and related agents on human chorionic gonadotropin secretion in human malignant trophoblast in culture. *In Vitro*, **13**, 443.

KNOTH, M., PATTILLO, R. A., GARANCIS, J. C., GEY, G. O., RUCKERT, A. C. F. & MATTINGLY, R. F. (1969) Ultrastructure and hormone synthesis of choriocarcinoma *in vitro*. *Am. J. Pathol.* **54**, 479.

KRAM, R., MAMONT, P. & TOMKINS, G. M. (1973) Pleiotypic control by cyclic AMP: a model for growth control in animal cells. *Proc. Natl. Acad. Sci., U.S.A.*, **70**, 1432.

PATTILLO, R. A., GEY, G. O., DELFS, E., HUANG, W. Y., HAUSE, L., GARANCIS, J. & 5 other authors. (1969) The hormone-synthesizing trophoblastic cell *in vitro*: A model for cancer research and placental hormone synthesis. *Ann. N.Y. Acad. Sci.*, **172**, 288.

ROBISON, G. A., BUTCHER, R. W. & SUTHERLAND, E. W. (1968) Cyclic AMP. *Annu. Rev. Biochem.*, **37**, 149.

STORY, M. T., HUSSA, R. O. & PATTILLO, R. A. (1974) Independent dibutyryl cyclic adenosine monophosphate stimulation of human chorionic gonadotropin and estrogen secretion by malignant trophoblast cells *in vitro*. *J. Clin. Endocrinol. Metab.*, **39**, 877.

WRIGHT, J. A. (1973) Morphology and growth rate changes in Chinese hamster cells in presence of sodium butyrate. *Exp. Cell Res.*, **78**, 456.